# Two new genera of nematode (Oxyurida, Hystrignathidae) parasites of Passalidae (Coleoptera) from the Democratic Republic of Congo

**DOI:** 10.3897/zookeys.257.3666

**Published:** 2013-01-04

**Authors:** Jans Morffe, Nayla García

**Affiliations:** 1 Instituto de Ecología y Sistemática, Carretera de Varona km 31/2, Capdevila, Boyeros, Habana 19, C.P.10800, La Habana, Cuba

**Keywords:** Nematoda, Hystrignathidae, *Kongonema*, *Lubanema*, Passalidae, Democratic Republic of Congo

## Abstract

Two new genera and species parasitizing passalid beetles from the Democratic Republic of Congo are described. *Kongonema meyeri* **gen. n. sp. n.** is characterized by having females with the cervical cuticle unarmed, first cephalic annule cone-like and truncate, sub-cylindrical procorpus and genital tract didelphic-amphidelphic. The males of *Kongonema meyeri* **gen. n. sp. n.** have the procorpus sub-cylindrical, the dorsal cuticle of the tail end thickened, a single large, median mammiform pre-cloacal papilla and a pair of small, pre-cloacal, sub-lateral papillae at a short distance before the level of the cloaca. *Lubanema decraemerae* **gen. n. sp. n.** is characterized by the body markedly fusiform, cuticle unarmed and strongly annulated, procorpus sub-cylindrical, isthmus as a constriction between procorpus and basal bulb, genital tract monodelphic-prodelphic and the posterior end rounded with a very short tail appendage.

## Introduction

The family Hystrignathidae comprises 27 nominal genera with more than 100 species of monoxenous nematodes specific of the hind gut of passalid beetles. The family shows a mostly Gondwanian distribution, with taxa from North, Central and South America, West Indies, Africa and Australasia (Adamson and Van Waerebeke 1992). Of these areas, the Americas and West Indies present the highest generic and specific diversity.

In Africa, the group still remains neglected with most of the species restricted to their type localities. Théodoridès (1955) described the first African hystrignathids: *Artigasia pauliani* Théodoridès, 1955and *Artigasia geopetiti* Théodoridès, 1955 from Malagasian passalids. Later, Théodoridès (1958) described *Artigasia pauliani* var. *joliveti* from the Democratic Republic of Congo. The status of this variety was analyzed by Adamson and Van Waerebeke (1992) who raised it to the rank of species.Baker (1967) recorded *Hystrignathus rigidus* Leidy, 1850 and *Xyo hystrix* Cobb, 1898 parasitizing three species of *Pentalobus* from Ghana. These two latter species had previously been described from North America and Australia, respectively. The main contribution to the knowledge of the family in the region was made by Van Waerebeke (1973), with the description of 14 species of *Artigasia* Christie, 1934 one of *Hystrignathus* Leidy, 1850and the monotypic genus *Passalidophila* Van Waerebeke, 1973 all from Madagascar. The author also re-described *Artigasia geopetiti* and recorded three types of males, unable to be assigned to their correct species. Van Waerebeke and Remillet (1982) described *Hystrignathus egalis* Van Waerebeke & Remillet, 1982 and *Hystrignathus inegalis* Van Waerebeke & Remillet, 1982 from Ivory Coast.

This paper retakes the study on African hystrignathids, describing two new genera and species parasitizing passalid beetles from the Democratic Republic of Congo.

## Material and methods

Several specimens of passalid beetles from the Democratic Republic of Congo (formerly Zaire) were examined in a parasitological survey during a research visit to the Royal Museum of Central Africa, Tervuren, Belgium. Eight specimens of *Didimus* sp. and two specimens of *Erionomus pilosus* Aurivillus, 1896 from Katale, Kivu region were included in this study, all collected during the Belgian expeditions to the Congo in the 1930´s and stored in 70% ethanol.

The hosts were dissected by practicing incisions in both pleural membranes and the intestines were extracted and kept in Petri dishes with 70% ethanol. The guts were excised and the parasites removed.

Nematodes were transferred and cleared in glycerine via slow evaporation method (Seinhorst 1959) and mounted in the same medium. The edges of the coverslips were sealed using nail polish. Measurements were made with a calibrated eyepiece micrometer attached to a compound microscope. De Man’s ratios a, b, c and V% were calculated. Each variable is shown as the range followed by the mean plus standard deviation in parentheses; the number of measurements is also given. Micrographs were taken with an AxioCam digital camera attached to a Carl Zeiss AxioScop 2 Plus compound microscope. Line drawings were made with the softwares CorelDRAW X3 and Adobe Photoshop CS2 using the micrographs as templates. Scale bars of all plates are given in millimeters.

Some specimens were prepared for SEM as follows: they were dehydrated in a graded ethanol series, critical point-dried, mounted in aluminum stubs and coated in gold. SEM micrographs were taken at an acceleration voltage of 22–25 kV.

Classification at generic level was followed after Adamson and Van Waerebeke (1992). For comparison, one paratype of *Passalidophila exceptionalis* Van Waerebeke, 1973; deposited in the Nematode Collection of the Museum of Natural History, Paris (MNHN) was reviewed. The type material and vouchers of the next taxa are deposited in the Colección Helmintológica de las Colecciones Zoológicas (CZACC), Instituto de Ecología y Sistemática, Havana, Cuba; the Collection of the Royal Museum of Central Africa (RMCA), Tervuren, Belgium; the Royal Belgian Institute of Natural Sciences (RBINS), Brussels, Belgium and the Coleçao Helmintologica do Instituto Oswaldo Cruz (CHIOC), Rio de Janeiro, Brazil.

## Systematics

### Family Hystrignathidae Travassos, 1920

#### Genus Kongonema gen. n.

urn:lsid:zoobank.org:act:0D693E9A-DB4B-4740-92FA-2053B0F574AC

http://species-id.net/wiki/Kongonema

##### Generic diagnosis.

**Female.** Body comparatively robust. Cervical cuticle unarmed. Lateral alae present, from the oesophageal region to a short distance beyond the level of the anus. Posterior ends of the lateral alae rounded, forming lobes. Head bearing eight paired papillae. First cephalic annule cone-like, truncate, barely inflated, about two head-lengths long. Oesophagus consisting of a muscular sub-cylindrical procorpus, its base well set-off from the isthmus. Nerve ring encircling procorpus at its midpoint. Excretory pore post-bulbar. Reproductive system didelphic-amphidelphic. Eggs ovoid, ridged-shelled. Tail filiform and subulate.

**Male.** Body shorter and more slender than female. Cervical cuticle unarmed. Lateral alae present, from the oesophageal region to the level of the single median mammiform papilla. First cephalic annule inconspicuous. Stoma scarcely developed. Oesophagus with a sub-cylindrical procorpus, well set-off from the short isthmus. Nerve ring encircling procorpus at its posterior half. Excretory pore post-bulbar. Monorchic. Testis outstretched. Spicule absent. Posterior end ventrally curved, tapering abruptly, forming a very short, rounded tail appendage. Dorsal cuticle of the tail end thickened. A single large, median mammiform pre-cloacal papilla present. A pair of small, pre-cloacal, sub-lateral papillae located at a short distance before the level of the cloaca.

##### Type species.

*Kongonema meyeri* Morffe & García gen. n. sp. n. (monotypic genus).

##### Distribution.

Democratic Republic of Congo.

##### Etymology.

The generic name (neuter) is a combination of Kongo, after the main ethnic group in the country of this taxon, and the suffix –nema.

#### Kongonema meyeri sp. n.

urn:lsid:zoobank.org:act:0E02D195-40DE-4D6A-A752-D9FEAF8910E7

http://species-id.net/wiki/Kongonema_meyeri

**Figure 1.**
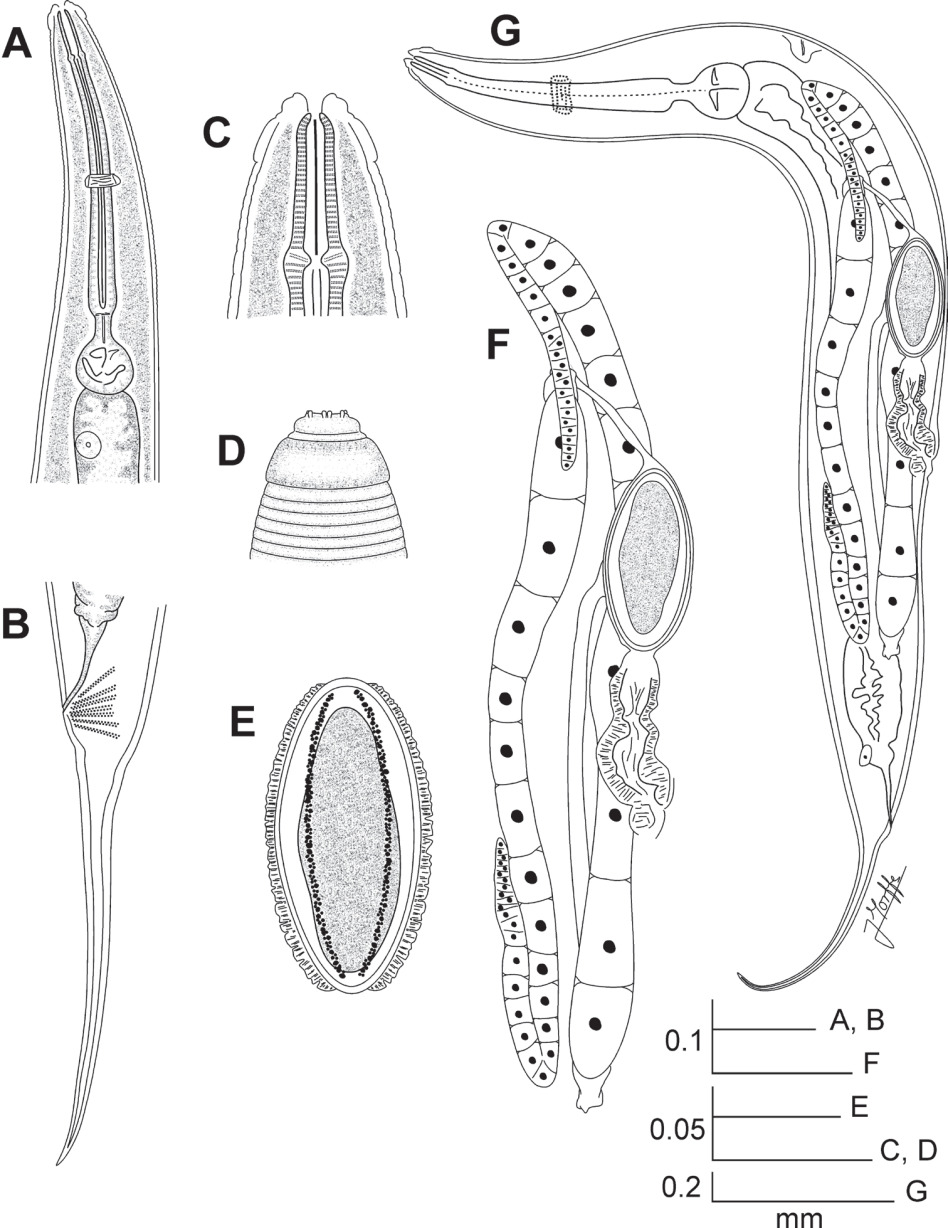
*Kongonema meyeri* gen. n. sp. n. Female. **A** Oesophageal region, ventral view **B** Tail, lateral view **C** Cephalic end, internal view **D** Cephalic end, external view **E** Egg **F** Reproductive system, lateral view **G** Entire nematode, lateral view.

**Figure 2.**
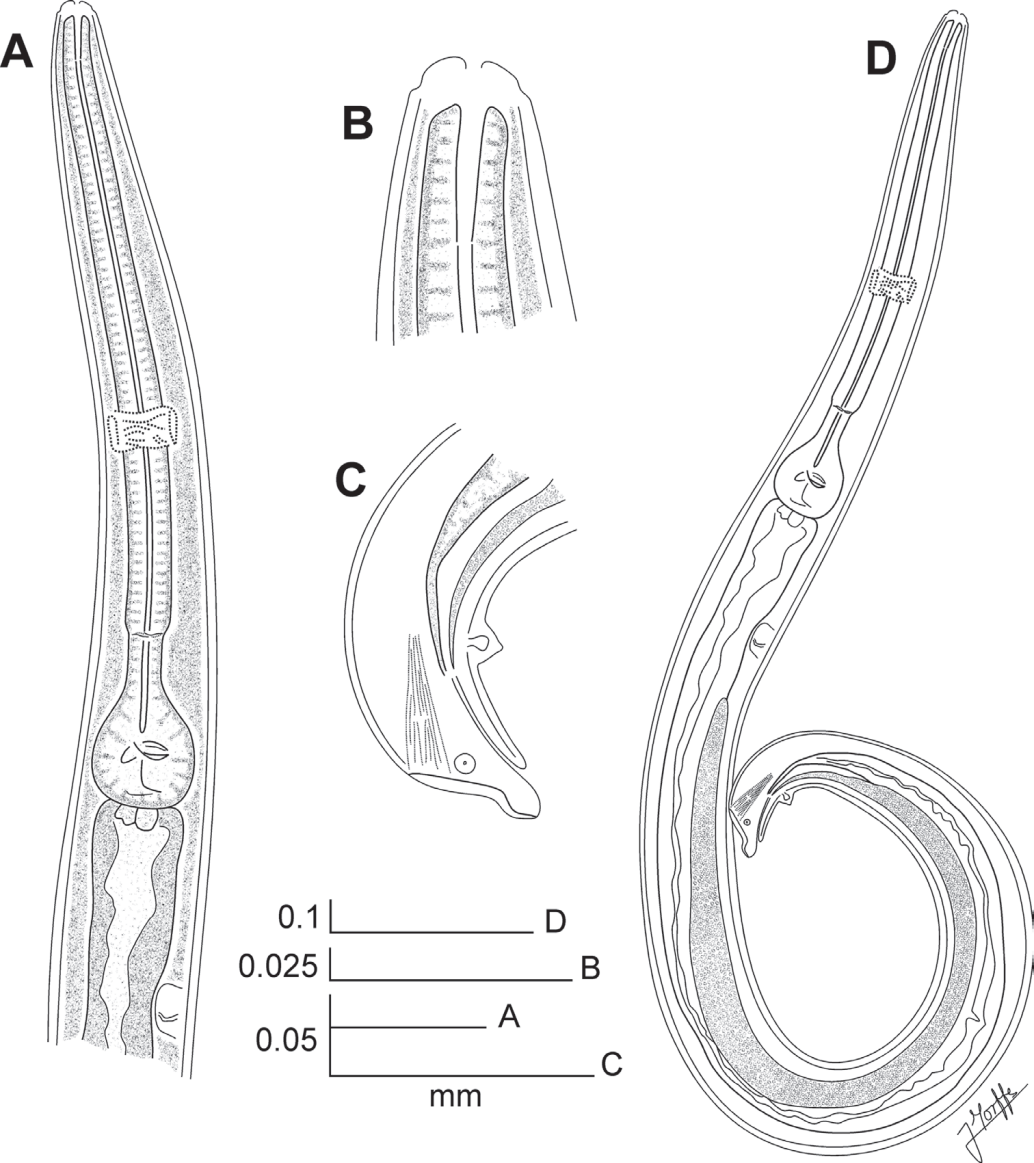
*Kongonema meyeri* gen. n. sp. n. Male. **A** Oesophageal region, lateral view **B** Cephalic end, internal view **C** Posterior end, lateral view **D** Entire nematode, lateral view.

**Figure 3.**
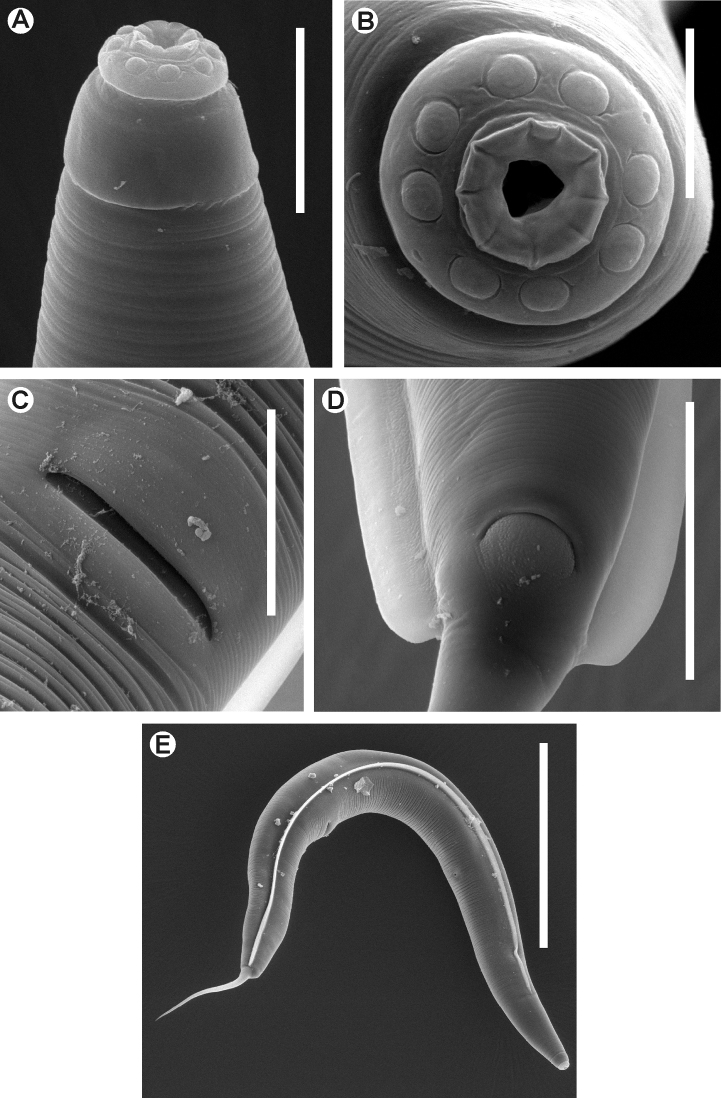
*Kongonema meyeri* gen. n. sp. n. Female. SEM images **A** Cephalic end **B** Cephalic end, *en face* view **C** Vulva **D** Anus and end of lateral alae **E** Habitus, lateral view. Scale lines: **A** 0.025 mm, **B** 0.01 mm, **C,** **D** 0.04 mm, **E** 0.3 mm.

Figs 1 A–G , 2 A–D , 3 A–E

##### Type material.

♀ holotype, Democratic Republic of Congo, Kivu Region, Katale, 1°19'S, 29°22'E; in *Didimus* sp.; 4.V.1939; Hautmann coll.; CZACC 11.4653. Paratypes: 10♀♀, same data as holotype, CZACC 11.4654-11.4663; 10♀♀, same data as holotype, RMCA; 4♀♀, same data as holotype, CHIOC; ♂, same data as holotype, CZACC 11.4664; ♂, same data as holotype, RMCA.

##### Additional material.

Vouchers: 2♀♀, Democratic Republic of Congo, Kivu Region, Katale, 1°19'S, 29°22'E; in *Didimus* sp.; 4.V.1939; Hautmann coll., RBINS. 2♀♀, Democratic Republic of Congo, Kivu Region, Katale, 1°19'S, 29°22'E; in *Erionomus pilosus*; 4.V.1939; Hautmann coll.;CZACC 11.4665-11.4666; 2♀♀, same data as the latter, RMCA;

##### Measurements.

Holotype (female) a = 12.15, b = 5.06, c = 7.26, V% = 58.08, total length = 1.670, maximum body width = 0.138, first cephalic annule (length×width) = 0.013×0.038, stoma length = 0.050, procorpus length = 0.260, isthmus length = 0.020, diameter of basal bulb = 0.058, total length of oesophagus = 0.330, nerve ring to anterior end = 0.185, excretory pore to anterior end = 0.440, vulva to posterior end = 0.700, anus to posterior end = 0.230, eggs = 0.123×0.050 (n = 1).

Paratypes (females) (n = 24) a = 8.65-13.08 (10.68 ± 0.90 n = 23), b = 4.30-5.03 (4.71 ± 0.71 n = 21), c = 5.65-7.45 (6.43 ± 0.37 n = 23), V% = 53.02-58.82 (55.91 ± 1.43 n = 23), total length = 1.400-1.670 (1.530 ± 0.075 n = 23), maximum body width = 0.120-0.170 (0.144 ± 0.012 n = 24), first cephalic annule (length×width) = 0.013-0.025×0.038-0.043 (0.016 ± 0.003×0.041 ± 0.002 n = 19), stoma length = 0.033-0.050 (0.045 ± 0.005 n = 19), procorpus length = 0.210-0.270 (0.244 ± 0.013 n = 20), isthmus length = 0.020-0.033 (0.024 ± 0.003 n = 22), diameter of basal bulb = 0.053-0.070 (0.061 ± 0.004 n = 24), total length of oesophagus = 0.283-0.350 (0.324 ± 0.014 n = 21), nerve ring to anterior end = 0.148-0.190 (0.172 ± 0.011 n = 21), excretory pore to anterior end = 0.320-0.490 (0.402 ± 0.046 n = 23), vulva to posterior end = 0.620-0.750 (0.674 ± 0.038 n = 23), anus to posterior end = 0.200-0.280 (0.239 ± 0.019 n = 23), eggs = 0.120-0.133×0.043-0.063 (0.125 ± 0.004×0.051 ± 0.006 n = 26).

Paratypes (males) (n = 2) a = 15.67-17.33 (16.50 ± 1.18 n = 2), b = 3.47-3.58 (3.52 ± 0.08 n = 2), c = 121.33-125.33 (123.33 ± 2.83 n = 2), total length = 0.910-0.940 (0.925 ± 0.021 n = 2), maximum body width = 0.053-0.060 (0.056 ± 0.005 n = 2), procorpus length = 0.250 (n = 2), isthmus length = 0.018-0.020 (0.019 ± 0.002 n = 2), diameter of basal bulb = 0.035 (n = 2), total length of oesophagus = 0.263 (n = 2), nerve ring to anterior end = 0.138-0.148 (0.143 ± 0.007 n = 2), excretory pore to anterior end = 0.290-0.330 (0.310 ± 0.028 n = 2), cloacae to posterior end = 0.008 (n = 2).

##### Specimens from *Erionomus pilosus*.

Females (n = 4) a = 9.46-10.75 (10.06 ± 0.68 n = 4), b = 4.75-4.97 (4.87 ± 0.09 n = 4), c = 6.58-7.13 (6.82 ± 0.23 n = 4), V% = 55.56-61.59 (57.75 ± 2.72 n = 4), total length = 1.640-1.750 (1.703 ± 0.046 n = 4), maximum body width = 0.153-0.185 (0.170 ± 0.015 n = 4), first cephalic annule (length×width) = 0.018-0.020×0.043-0.048 (0.019 ± 0.001×0.045 ± 0.002 n = 4), stoma length = 0.048-0.053 (0.050 ± 0.003 n = 4), procorpus length = 0.255-0.275 (0.267 ± 0.009 n = 4), isthmus length = 0.020-0.025 (0.023 ± 0.002 n = 4), diameter of basal bulb = 0.068-0.075 (0.071 ± 0.004 n = 4), total length of oesophagus = 0.330-0.360 (0.350 ± 0.014 n = 4), nerve ring to anterior end = 0.185-0.195 (0.189 ± 0.004 n = 4), excretory pore to anterior end = 0.420-0.510 (0.475 ± 0.040 n = 4), vulva to posterior end = 0.630-0.760 (0.720 ± 0.61 n = 4), anus to posterior end = 0.230-0.260 (0.250 ± 0.014 n = 4), eggs = 0.120-0.130×0.048-0.065 (0.126 ± 0.004×0.058 ± 0.007 n = 7).

##### Description.

**Female.**Body comparatively robust, widening from the base of the first cephalic annule, maximum body diameter at level of the vulva, then tapering towards anus. Cervical cuticle unarmed, markedly annulated (annuli *ca*. 5-7 µm). Rest of the body with marked annuli decreasing their width towards the level of the anus. Sub-cuticular longitudinal striae present. Lateral alae *ca*. 9 µm wide, from the oesophageal region (*ca*. 30 µm before the level of the nerve ring) to a very short distance beyond the level of the anus. Posterior ends of the lateral alae rounded, forming short lobes. Head well developed, set-off from body by a single, deep groove and bearing eight rounded, paired papillae. Amphids pore-like, laterally situated. Mouth sub-triangular in shape. First cephalic annule cone-like, truncate, barely inflated, about two head-lengths long. Stoma comparatively long, about three first cephalic annule lengths long, surrounded by an oesophageal collar. Oesophagus consisting of a muscular, sub-cylindrical procorpus, its base slightly wider and well set-off from the short isthmus. Basal bulb sub-spherical, valve plate well developed. Intestine simple, sub-rectilinear, anterior portion dilated. Rectum short, anus not prominent, as a crescent-like slit. Nerve ring encircling procorpus at about its midpoint. Excretory pore situated at about half of a body width posterior to basal bulb. Genital tract didelphic-amphidelphic, both ovaries reflexed. Anterior ovary reflexed behind the excretory pore, posterior ovary reflexed at about a body width before the anus. Distal flexures of ovaries about one body width-length long. Oöcytes in single rows. Vulva a median transverse slit slightly displaced to the posterior half of body, lips prominent. Vagina muscular, forwardly directed. Eggs ovoid, bearing eight longitudinal, rough, ridges on the shell. Tail comparatively long, filiform, subulate, ending in a sharp point.

##### Male.

Body shorter than female, comparatively slender, posterior region ventrally curved. Cervical cuticle unarmed. Sub-cuticular longitudinal striae present. Lateral alae from the oesophageal region (about three body-widths posterior to the cephalic end) to the level of the single mammiform papilla (about a body-width before the level of anus). Head not set-off from body. First cephalic annule not developed. Stoma not defined. Oesophagus consisting of a sub-cylindrical procorpus, well set-off from the short isthmus. Basal bulb rounded, valve plate well developed. Intestine simple, anterior portion slightly dilated. Nerve ring encircling procorpus at its posterior half, about 65% of its length. Excretory pore situated at about 1.5 body-widths posterior to basal bulb. Monorchic. Testis outstretched, arising at a short distance behind the excretory pore. Spicule absent. Dorsal cuticle of the tail region thickened. A single, large, pre-cloacal ventromedian mammiform papilla situated at about a body width before the posterior end. A pair of small, pre-cloacal, sub-lateral papillae situated at a short distance before the level of the cloaca. Tail region becoming sharp visibly from the beginning of the cuticular thickening, until forming a very short tail appendage, its tip rounded.

##### Discussion.

There are three genera of hystrignathids the female of which present the cervical cuticle unarmed, procorpus sub-cylindrical and reproductive system digonant: *Anomalostoma* Cordeira, 1981; *Coynema* (Coy, García & Alvarez, 1993) Morffe & García, 2011 and *Ventelia* Travassos & Kloss, 1958. The first differs by having the anterior region of the procorpus strongly swollen, surrounding the stoma (Cordeira 1981). The stoma of *Kongonema* gen. n. is surrounded only by an oesophageal collar, as occur in many hystrignathids. *Anomalostoma* lacks an evident first cephalic annule *vs*. conspicuous first cephalic annule of *Kongonema* gen. n.

Females of *Coynema* can be segregated by the basal dilatation of its procorpus and the anterior region of the intestine notably inflated, forming a saccular structure (Morffe and García 2011). Both traits are absent in *Kongonema* gen. n., which procorpus increases its diameter slightly and gradually towards its base and the fore region of the intestine is only moderately inflated, without the saccular structure mentioned above. The oviduct next to the vagina forms a loop in *Coynema*, instead of the straight oviduct of the present genus.

The males of *Kongonema* gen. n. resemble their counterparts of *Coynema* (only close genus where the male is known) by lacking of spicule and by having a similar arrangement of the copulatory papillae: the ventromedian pre-cloacal papilla (typical of Hystrignathidae) and another pair of small sub-lateral pre-cloacal papillae. *Kongonema* gen. n. differs by having a sub-cylindrical procorpus, without the basal dilation and by lacking the saccular region of the intestine characteristic of *Coynema*. The posterior end of *Kongonema* gen. n. forms a short, rounded tail appendage *vs*. the sharp tail of *Coynema*.

On the other hand, *Ventelia* has the procorpus barely set-off from the isthmus, since the posterior third of the procorpus decreases its diameter. The hind procorpus of *Kongonema* gen. n. increases its diameter slightly and is well differentiated from the isthmus.

##### Type host.

*Didimus* sp. (Coleoptera: Passalidae).

##### Other host.

*Erionomus pilosus* Aurivillus, 1896 (Coleoptera: Passalidae).

##### Site.

Gut caeca.

##### Type locality.

Katale, Kivu region, Democratic Republic of Congo.

##### Etymology.

Specific epithet dedicated to Dr. Marc de Meyer, curator of the Entomological Collection of the Royal Museum of Central Africa, Tervuren, Belgium. In appreciation of his kind help by permitting access to the material assessed.

#### Genus Lubanema gen. n.

urn:lsid:zoobank.org:act:F73361A0-2822-4BAE-8976-05BA66452B9E

http://species-id.net/wiki/Lubanema

##### Generic diagnosis.

**Female.**Body notably robust and fusiform. Posterior end strongly rounded, bearing a terminal, very short, conical tail appendage. Cuticle unarmed, markedly annulated until the level of the anus. Lateral alae wide, from the oesophageal region to the level of the anus. Posterior ends of the alae almost forming a straight angle with the body axis, slightly convex and with short lobes in their margins. First cephalic annule cone-like, slightly inflated, its margins convex. Oesophagus with a sub-cylindrical, muscular procorpus. Isthmus as a constriction between the procorpus and basal bulb. Nerve ring encircling procorpus at its posterior half. Excretory pore post-bulbar. Reproductive system monodelphic-prodelphic. Ovary stout. Eggs markedly ovoid, smooth-shelled.

##### Type species.

*Lubanema decraemerae* Morffe & García gen. n. (monotypic genus).

##### Distribution.

Democratic Republic of Congo.

##### Etymology.

The generic name (neuter) is a combination of Luba, after one of the ethnic groups in the country, and the suffix –nema.

#### Lubanema decraemerae sp. n.

urn:lsid:zoobank.org:act:D480D872-86F3-4920-B9A7-550925CCD97A

http://species-id.net/wiki/Lubanema_decraemerae

Figs 4 A–G , 5 A–D

##### Type material.

♀ holotype, Democratic Republic of Congo, Kivu Region, Katale, 1°19'S, 29°22'E; in *Didimus* sp.; 4.V.1939; Hautmann coll.; CZACC 11.4667. Paratypes: ♀, same data as holotype, CZACC 11.4668; ♀, same data as holotype, RMCA.

##### Measurements.

Holotype (female) a = 6.70, b = 5.97, c = 40.18, V% = 57.92, total length = 2.210, maximum body width = 0.330, first cephalic annule (length×width) = 0.020×0.070, stoma length = 0.048, procorpus length = 0.268, diameter of basal bulb = 0.108, total length of oesophagus = 0.370, excretory pore to anterior end = 0.520, vulva to posterior end = 0.930, anus to posterior end = 0.055, eggs = 0.168-0.173×0.075-0.080 (0.170 ± 0.004×0.078 ± 0.004 n = 2).

Paratypes (females) (n = 2) a = 4.44-6.36 (5.40 ± 1.36 n = 2), b = 3.66-5.68 (4.67 ± 1.43 n = 2), c = 34.45-38.18 (36.31 ± 2.64 n = 2), V% = 56.67 (n = 1), total length = 2.100-2.400 (2.250 ± 0.212 n = 2), maximum body width = 0.330 (n = 2), first cephalic annule (length×width) = 0.020×0.063-0.065 (0.020×0.064 ± 0.002 n = 2), stoma length = 0.043-0.045 (0.044 ± 0.002 n = 2), procorpus length = 0.275-0.300 (0.288 ± 0.018 n = 2), diameter of basal bulb = 0.108 (n = 2), total length of oesophagus = 0.370-0.400 (0.385 ± 0.021 n = 2), nerve ring to anterior end = 0.223 (n = 1), excretory pore to anterior end = 0.410-0.600 (0.505 ± 0.134 n = 2), vulva to posterior end = 1.040 (n = 1), anus to posterior end = 0.043-0.055 (0.049 ± 0.009 n = 2), eggs = 0.183×0.078 (n = 1).

##### Description.

Female body large, notably robust and fusiform, widening gradually from the base of the first cephalic annule, reaching maximum width near mid-body, then tapering softly towards the posterior end that rounds off abruptly. A comparatively very short, conical tail appendage with its tip rounded arises terminally from the posterior end. Cervical cuticle unarmed, with marked annule (*ca*. 13 µm wide), extending to the rest of body, until level of the anus. Lateral alae thick, *ca*. 55 µm wide, extending from the hind third of the procorpus to the level of the anus. Posterior ends of lateral alae almost forming a straight angle with the body axis, slightly convex, their external margins forming a very short lobe. Head bearing eight rounded, paired papillae, set-off from body by a single, deep groove. Amphids pore-like, laterally situated. Mouth trirradiate. First cephalic annule cone-like, slightly inflated, its margins convex, about two head-lengths long. Stoma long, about 1.5 first cephalic annule lengths long, surrounded by an oesophageal collar. Oesophagus consisting of a muscular, sub-cylindrical procorpus, its diameter little increased at its base. Isthmus as a constriction between the procorpus and the large, rounded, basal bulb. Valve plate well developed. Intestine simple, sub-rectilinear, its fore region very inflated. Rectum short. Anus sub-terminal. Nerve ring encircling procorpus at its posterior half (*ca*. 60% of its length). Excretory pore located at about the half of a body width behind the basal bulb. Vulva a median trasverse slit, displaced to the posterior half of body, lips less prominent. Vagina muscular, forwardly directed. Genital tract monodelphic-prodelphic. Ovary stout, reflexed at about one third of the body width behind the basal bulb. Oöcytes in a single row, about four times wider than long (*ca*. 8×2 µm). Eggs large, markedly ovoid, smooth-shelled. Male unknown.

**Figure 4.**
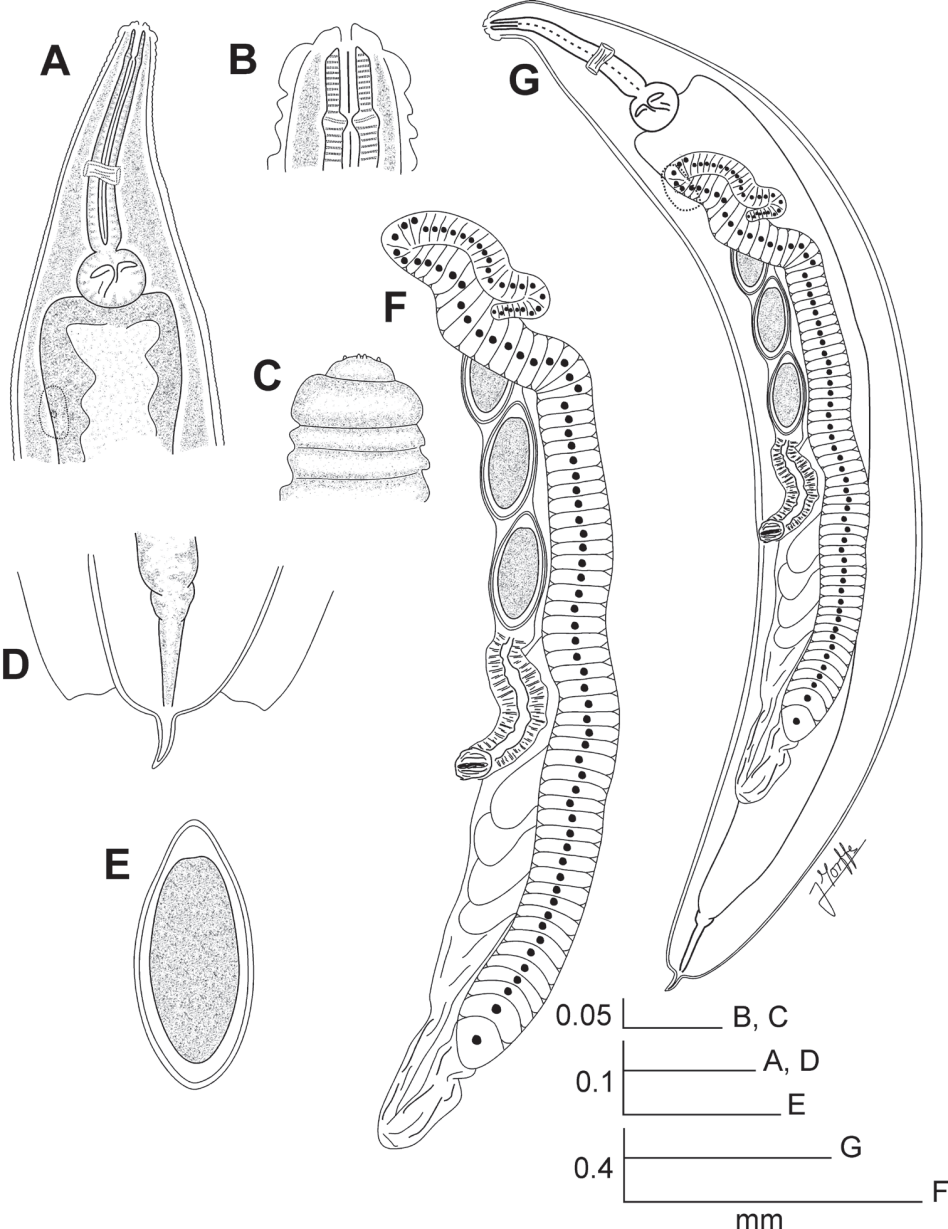
*Lubanema decraemerae* gen. n. sp. n. Female. **A** Oesophageal region, ventrolateral view **B** Cephalic end, internal view **C** Cephalic end, external view **D** Tail, ventral view **E** Egg **F** Reproductive system, ventrolateral view **G** Entire nematode, ventrolateral view.

**Figure 5.**
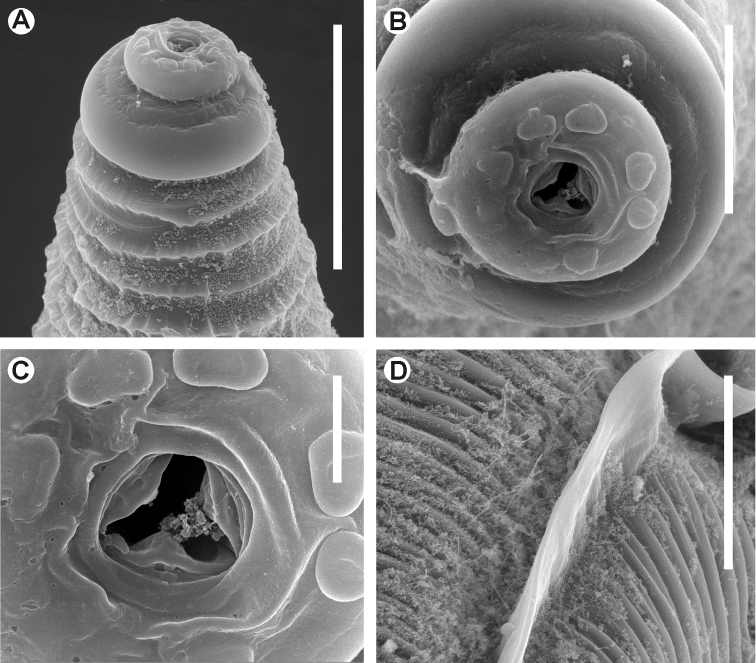
*Lubanema decraemerae* gen. n. sp. n. Female. SEM images. **A** Cephalic end **B** Cephalic end, *en face* view **C** Mouth **D** Lateral ala, detail. Scale lines: **A,** **D** 0.05 mm, **B** 0.02 mm, **C** 0.005 mm

##### Discussion.

The Malagasian genus *Passalidophila* resembles *Lubanema* gen. n. by having both the body robust and fusiform, cervical cuticle unarmed and markedly annulated, a similar form of the cephalic end, the lateral alae extending from the level of the procorpus to the anus and the tail short (Van Waerebeke 1973). Differs by having a procorpus which diameter increases towards its joint with the isthmus. *Lubanema* gen. n. have a more cylindrical procorpus and the isthmus is absent. The tail of *Passalidophila* is subulate, instead of the current new genus, which presents a very short tail appendage arising from the rounded posterior end. In addition, the ovary of *Passalidophila* is slender *vs*. the robust ovary of *Lubanema* gen. n.

Other monogonant hystrignathid genera with smooth cervical cuticle are *Christiella* Travassos & Kloss, 1957; *Coronocephalus* Cordeira, 1981; *Glaber* Travassos & Kloss, 1958; *Longior* Travassos & Kloss, 1958; and *Vulcanonema* Travassos & Kloss, 1958. All of these taxa can be differentiated from *Lubanema* gen. n. by having a well developed tail, from attenuate to subulate. *Christiella* and *Longior* females have a comparatively slender body *vs*. the notably more robust and fusiform body of *Lubanema* gen. n. Also, both genera present cylindrical procorpus more elongate than in *Lubanema* gen. n.

*Coronocephalus* bears prominent, digitiform oral papillae, instead of the shorter, less developed papillae of *Lubanema* gen. n. In the latter genus the procorpus meets directly the basal bulb, while *Coronocephalus* present an isthmus. *Glaber* differs from *Lubanema* gen. n. by having the base of the procorpus clavate, instead of the sub-cylindrical procorpus present in the new genus.

*Vulcanonema* presents the cephalic end consisting of a narrow cephalic annule separated of the head by a conical region. In opposition, *Lubanema* gen. n. have the first cephalic annule just after the head. Also, the procorpus of *Vulcanonema* is sub-cylindrical, with a basal dilation, absent in the present new genus.

*Lubanema* gen. n. shows morphological affinities with the Australian genera *Anuronema* Clark, 1978 and *Sprentia* Clark, 1978 by having the cuticle unarmed and strongly annulated, reduction of the isthmus and the tail. Moreover, the lateral alae of *Anuronema* extends from the oesophageal region to almost the level of the anus, similar to *Lubanema* gen. n. The new genus differs from both by its genital tract monodelphic-prodelphic *vs*. didelphic-amphidelphic, procorpus sub-cylindrical *vs*. claviform and development of the lateral alae, which in *Lubanema* gen. n. are very wide and with lobes in the margins at their terminal ends.The procorpus is widely amalgamated with the basal bulb in *Anuronema* and *Sprentia*, whereas *Lubanema* gen. n. presents a well defined constriction separating both structures. *Anuronema* has a total reduction of the tail appendage (Clark 1978) not observed in *Lubanema* with a short, conical tail.

*Carlosia* Travassos & Kloss, 1957 also presents a reduction of the isthmus, a large, slightly inflated first cephalic annule and marked annule in the cervical region (Hunt 1982). It can be easily segregated from *Lubanema* gen. n. by having a didelphic-amphidelphic genital tract and the annule of the cervical cuticle retrorse, with posterior prolongations forming a double row of spines laterally situated.

##### Type host.

*Didimus* sp. (Coleoptera: Passalidae).

##### Site.

Hind gut, out of the caeca.

##### Type locality.

Katale, Kivu region, Democratic Republic of Congo.

##### Etymology.

Specific epithet dedicated to Prof. Dr. Wilfrieda Decraemer, from the Royal Belgian Institute of Natural Sciences. In appreciation for her help and support during the current research.

## Supplementary Material

XML Treatment for Kongonema

XML Treatment for Kongonema meyeri

XML Treatment for Lubanema

XML Treatment for Lubanema decraemerae
